# Self-Surfactant Poly-3hydroxybutyrate-*co*-3hydroxyhexanoate (PHBHHx) for the Preparation of Usnic Acid Loaded Antimicrobial Nanoparticles Using Nontoxic Chemicals

**DOI:** 10.1021/acsabm.5c00676

**Published:** 2025-06-19

**Authors:** Sara Alfano, Lorenzo Ceparano, Benedetta Brugnoli, Gianluca Forcina, Luca Pellegrino, Francesca Cecilia Lauta, Roberto Rusconi, Iolanda Francolini, Antonella Piozzi, Andrea Martinelli

**Affiliations:** a Department of Chemistry, 9311 Sapienza University of Rome, P.le A. Moro 5, Rome 00185, Italy; b Department of Biomedical Sciences, 437807 Humanitas University, Via Rita Levi Montalcini 4, Pieve Emanuele 20072, Italy; c IRCCS Humanitas Research Hospital, Via Manzoni 56, Rozzano 20089, Italy

**Keywords:** polyhydroxyalkanoates, choline taurinate, sustainability, usnic acid, antimicrobial, antifouling, microfluidics

## Abstract

Polyhydroxyalkanoates (PHAs) are naturally occurring polyesters with promising drug delivery applications. Their hydrophobicity enables lipophilic drug encapsulation, enhancing bioavailability but limiting colloidal stability and physiological compatibility. Surfactants crucially improve the nanoparticle dimensional stability, dispersion, wettability of hydrophobic matrices, and cellular interaction, yet conventional surfactants require additional purification and may pose physiological risks. Self-surfactant systems offer a sustainable alternative. Therefore, this research proposes a green chemical modification of PHAs to develop self-surfactant systems. Hydrophilic groups were introduced onto a poly-3-hydroxybutyrate-*co*-3-hydroxyhexanoate (PHBHHx) backbone via amidation using choline taurinate ([Ch]­[Tau]), a biocompatible ionic liquid. This approach eliminates the need for toxic reagents and complex purification. By precisely controlling the PHBHHx/[Ch]­[Tau] molar ratio, amphiphilic structures with varying hydrophobic tail lengths were produced, as confirmed by infrared spectroscopy and chromatographic analysis. Nanoparticles were fabricated through the emulsion-solvent evaporation method and employed to encapsulate the lipophilic and antimicrobial agent usnic acid. Dynamic light scattering highlighted the obtainment of stable colloidal suspensions with dimensions of 40–160 nm. Biological evaluations demonstrated the antimicrobial efficacy against planktonic Newman strain and biofilm inhibition under fluidic conditions even for the unloaded nanoparticles. Additionally, the nanoparticles exhibited no cytotoxicity at concentrations ranging from 10 to 0.1 μg/mL while retaining antimicrobial activity, in contrast to the high cytotoxicity observed for free usnic acid. Overall, this approach offers a sustainable and scalable strategy to produce self-surfactant and intrinsically antimicrobial polymeric nanocarriers suitable for the systemic drug delivery of lipophilic compounds, smart implant coatings, and antibacterial topical formulations.

## Graphical abstract

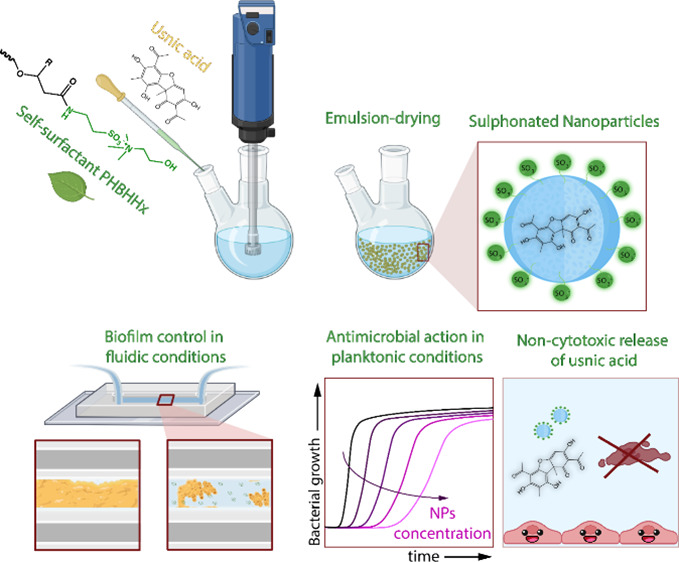

## Introduction

Over the past few years, the use of natural substances for biomedical applications gained considerable interest because of their anti-inflammatory, antimicrobial, antioxidant, and, in some cases, anticancer properties as for curcumin,^1,2^ usnic acid,^3–5^ and thymol.^6,7^ Despite their great therapeutic potential, the lipophilic nature of bioactive phytochemicals^8^ compromises their compatibility with the aqueous physiological environment. Consequently, they exhibit low bioavailability and stability and can trigger adverse immune reactions, thus limiting their clinical use. To overcome solubility-related issues, bioactive molecules might be encapsulated in a suitable nanosized carrier made of a biodegradable and biocompatible material. In this way, the lipophilic substance is protected and vehiculated through the aqueous environment while higher solubility, bioavailability, and lower side effects are ensured. Many examples of delivery nanosystems such as cyclodextrin complexes^9^ and lipid and polymeric nanoparticles (LNPs and PNPs, respectively)^10–12^ have been studied. PNPs are highly attractive for drug delivery because of the possibility to obtain controlled and targeted release by introducing functional groups able to interact with specific receptors.^13–16^ In this regard, polyesters are widely used materials owing to their inherent hydrophobicity, which confers a high affinity for lipophilic drugs. However, this characteristic hampers the polymer colloidal dispersion and interaction with cells. Surfactants play an essential role in stabilizing polyester NP dispersions, preventing aggregation, and promoting a uniform size distribution. However, the difficult excess removal, environmental concerns,^17,18^ cases of hypersensitivity, and suspected carcinogenetic effects encouraged the development of safer alternatives. Materials with intrinsic surfactant activity represent a promising approach. This class of materials, usually called self-surfactants, consist of amphiphilic molecules that self-assemble into micro- or nanostructures. The resulting systems are composed of hydrophobic cores decorated with hydrophilic moieties on the surface. The direct surfactant-free preparation of nanoparticles can involve a heterophase reaction (emulsion or suspension polymerization) where monomers are polymerized in the presence of amphiphilic comonomers, also known as surfmers. This approach results in stable suspensions of nanoparticles decorated with polar or charged groups.^19–21^

Ring-opening polymerization (ROP) is a bottom-up approach for synthesizing polyesters initiated by hydrophilic molecules or macromolecules. This method is commonly used to produce block copolymers with a hydrophobic polyester segment (e.g., polylactide, polyglycolide, or polycaprolactone) and a hydrophilic component composed of polyester-polyacrylate block copolymers^22^ or, more frequently, poly­(ethylene glycol) (PEG). ROP can also be initiated by small molecules such as 2,2-bis (hydroxymethyl) propionic acid,^23^ saccharide-based molecules,^24,25^ and *N*,*N*′-bis­(2-hydroxyethyl)­methylamine ammonium propanesulfonate.^26^ However, the solubility and dispersion limitations of polar or charged initiators may hinder the polymerization efficiency.

Alternatively, a “top-down” strategy is a valuable approach to modify preformed polyesters like those extracted from natural sources. Polar and/or ionic groups are introduced onto pre-existing polyester chains through various functionalization reactions.^27–29^ Among these, aminolysis has been widely used to endow polyester films with specific functional groups, enhancing hydrophilicity or preparing surfaces for grafting bioactive molecules to ameliorate the interaction with biological tissues or cells.^30–33^

While aminolysis is highly effective, it presents challenges related to safety and sustainability. The commonly used nucleophilic agents (e.g., hexamethylene diamine or ethylene diamine) and solvents (e.g., methanol and propanol) are toxic to living systems and ecosystems. Consequently, time-consuming purification steps are required to prevent the persistence of dangerous unreacted dangerous compounds. Recently, the use of naturally occurring substances as aminolysis reagents has been investigated. Pellegrino et al.^34^ studied the possibility of using the physiological amino acid taurine (Tau) for PLLA surface aminolysis, transforming Tau in its corresponding salts with tetrabutylammonium (TBA), but traces of TBA remained on the PLLA surface potentially causing low cell viability. To avoid negative effects on cell proliferation, De Felice et al.^35^ proposed an alternative, replacing the Tau counteranion with choline (Ch). The resulting ionic liquid, choline taurinate ([Ch]­[Tau]), showed no cytotoxicity and enabled the aminolysis of PLLA, which was subsequently used to prepare porous scaffolds.

The present work explored the use of [Ch]­[Tau] as a sustainable aminolysis reagent to synthesize amphiphilic polyhydroxyalkanoate (PHA) derivatives in the homogeneous phase. PHAs, like other polyesters, are biodegradable, bioresorbable, and nonimmunogenic.^36^ Specifically, the copolymer poly­(3-hydroxybutyrate-*co*-3hexanoate) (PHBHHx) was subjected to an aminolysis reaction with [Ch]­[Tau]. All synthetic procedures were designed to avoid time-consuming and complex purification stages. Ethanol and ethyl acetate were chosen as safe solvents,^37,38^ and the reaction was conducted in mild experimental conditions. The modified PHBHHx was used as a self-surfactant to prepare an oil-in-water emulsion to encapsulate usnic acid (UA). This dibenzofuran, originally isolated from lichens, is well-known for its antimicrobial, antiviral, antiproliferative, and anti-inflammatory activity but is poorly soluble in water.^39,40^ The characterization of obtained amphiphilic structures and nanoparticles was carried out by Fourier transform infrared spectroscopy (FTIR), dynamic light scattering (DLS), and gel permeation chromatography (GPC). The amount of UA encapsulated in NPs was evaluated by UV–vis spectroscopy.

The biological activity of pristine PHBHHx nanoparticles and nanoparticles loaded with usnic acid (PHBHHx-UA) was investigated by testing their antimicrobial activity and cytotoxicity. Specifically, the antimicrobial activity of PHBHHx was assessed using a clinically relevant strain, (strain Newman), which is involved in pathogenic infections and is known for its high virulence and antibiotic resistance.^41^ The impact of different nanoparticle formulations on the planktonic growth of was tested. Furthermore, the long-term antibiofilm formation was evaluated in microfluidic devices mimicking physiological conditions. The biocompatibility of PHBHHx and PHBHHx-UA nanoparticles was assessed by evaluating the viability, cytotoxicity, and apoptosis of pulmonary epithelial cells (A549) over 24 h.

By integrating chemical safety, sustainability, and tunability, the developed original method yields polymeric nanocarriers possessing the dual functionality of self-surfactancy and antimicrobial activity. This combination offers promising avenues for effective antibacterial topical applications and, prospectively, also for systemic delivery of other lipophilic drugs and smart implant coatings.

## Results and Discussion

The scheme of the aminolysis reaction of PHBHHx with [Ch]­[Tau] is reported in Figure 1.

**Figure 1:**
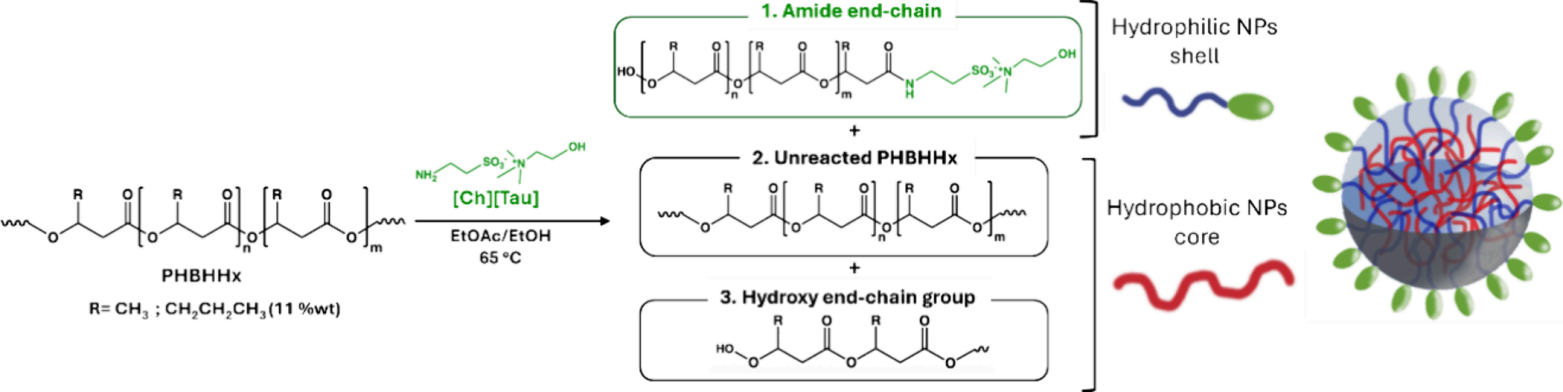
Aminolysis reaction. Schematic representation of the aminolysis reaction of PHBHHx using [Ch]­[Tau] at 65 °C. The reaction product presumably is a mixture of (1) aminolyzed PHBHHx with surfactant activity, (2) unreacted PHBHHx with high hydrophobicity, and (3) a hydroxy end-chain group.

The reaction involves the cleavage of an ester group and the formation of an amide bond by the primary amine of the ionic liquid, acting as a nucleophile. As a result, the −SO_3_^–^ group from the ionic liquid is introduced at one end of the cleaved chain, and a hydroxyl group is generated at the other. If aminolysis occurs randomly along the macromolecule, the products will be a mixture of shorter PHBHHx chains terminated with – SO_3_^–^ (constituting the external part of nanostructures in water suspension) and OH groups and presumably unreacted macromolecules (constituting the hydrophobic core of nanostructures in water suspension), exhibiting a wide distribution of molecular weights.

Many studies have reported the strong influence of reaction time on aminolysis.^46–49^ However, these studies are limited to heterogeneous reactions for surface functionalization. Therefore, the influence of different reaction times during homogeneous aminolysis has been performed. Preliminary experiments were conducted at 1, 2, and 4 h using an ionic liquid mole fraction of 0.05 corresponding to 1 mol of [Ch]­[Tau] each 20 mol of PHBHHx repeating units (RU). The results (Figure S1, Supporting Information) show that the *M*_n_ reached a constant value after 2 h, which was used in subsequent aminolysis experiments.

### Evaluation of the Effect of Different *X*_[Ch][Tau]_ on Aminolysis Products

PHBHHx-based self-surfactant systems with modulated hydrophobic/hydrophilic balances were synthesized by varying the RU to [Ch]­[Tau] ratio. More in detail, 1 mol of ionic liquid each of 2.5, 5, 10, and 20 mol of PHBHHx RU was used. For each condition, the reaction was carried out in duplicate.

The aminolysis reaction caused a reduction in the molecular weight of the polymer, as shown by the shift and the broadening of the GPC peak (Figure 2 A).

**Figure 2:**
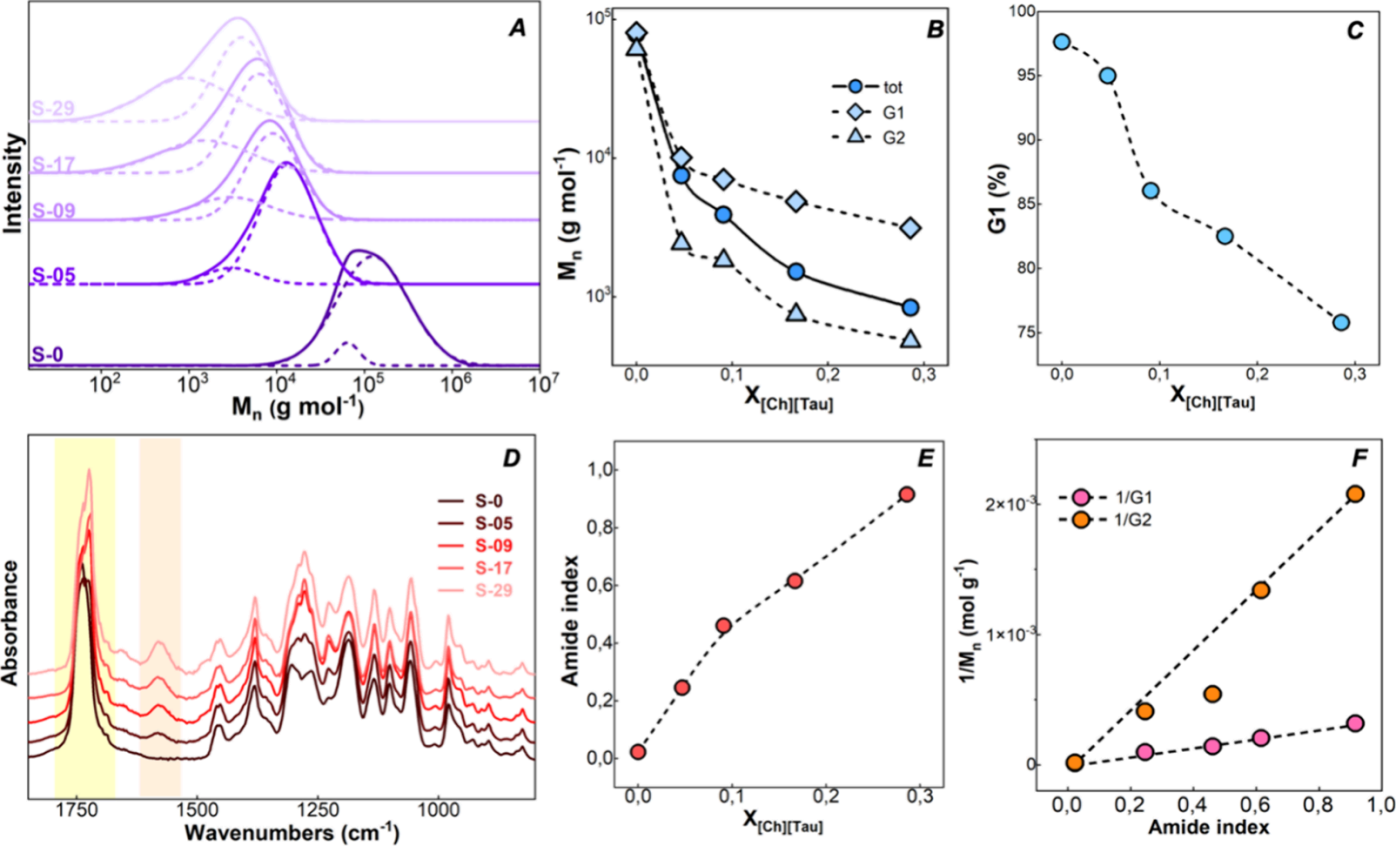
Amidated PHBHHx characterization. GPC and FTIR characterization of aminolysis products. (A) GPC chromatograms of pristine and aminolyzed PHBHHx by using different *X*_[Ch][Tau]_. Dotted lines are the Gaussian curves used to interpolate the chromatograms. (B) Number-average molecular weight of the aminolyzed sample as a function of *X*_[Ch][Tau]_ calculated from overall chromatograms (tot) and from the two interpolating Gaussian curves (G1 and G2). (C) Integrated area of the Gaussian function at the highest molecular weight G1 with respect to the overall chromatogram (G1%). (D) FTIR spectra of amidated PHBHHx prepared by using different [Ch]­[Tau] molar fractions *X*_[Ch][Tau]_ compared with pristine PHBHHx. (E) Variation of the amide index as a function of *X*_[Ch][Tau]_. (F) A linear relationship between the inverse of *M*_n_, calculated from G1 and G2, and the amide index.

Figure 2B demonstrates that even at the lowest aminolyzing agent concentration, a significant reduction in polymer molecular weight was observed compared to the pristine sample. This substantial decrease suggests that a large proportion of the polymer chains are actively involved in the aminolysis reaction. As a result, the polydispersity index (PDI) increased with *X*_[Ch][Tau]_, passing from 2.0 ± 0.03 of the S-0 sample to 2.2 ± 0.004, 2.5 ± 0.004, 3.1 ± 0.02, and 3.2 ± 0.007 for S-05, S-09, S-17, and S-29, respectively. This entails obtaining aminolyzed polymers with a progressively wider chain length distribution and good reproducibility, as the low standard deviation values testify. Moreover, all the chromatograms display a double distribution, clearly visible from the shoulder in correspondence with lower molecular weights. Then, the GPC curves were interpolated by the sum of two Gaussian functions (G1 and G2). The *M*_n_ values calculated by considering the overall chromatograms and single Gaussian curves are reported in Figure 2B. Figure 2C displays the integrated area of the Gaussian function (*A*_G1_) at the highest molecular weight, calculated according to eq 2. It shows that the shorter chain fraction is largely responsible for the observed reduction in overall molecular weight.

Changes in the PHBHHx chemical structure were analyzed by FTIR spectroscopy and NMR measurements (Figures S2–S5). Figure 2D shows the FTIR spectra of pristine PHBHHx and aminolyzed products obtained by using different [Ch]­[Tau] mole fractions. The spectra, normalized to the 1380 cm^–1^ absorption band (symmetrical CH_3_ deformation unaffected by the reaction), clearly show the appearance of amide bands at 1653 (amide I) and 1579 cm^–1^ (amide II). The effect of increasing the aminolyzing agent mole fraction on amide II absorbance was quantified using the amide index, reported as a function of *X*_[Ch][Tau]_ in Figure 2E. A notable linear correlation was observed between the amide index and the inverse of *M*_n_, a parameter indicative of macromolecule concentration and therefore also related to the concentration of amide groups (Figure 2F).

The aminolysis reaction was expected to yield taurine- and hydroxy-terminated chains. These products were then fractionated based on their differing solubilities. EtOH was added to a chloroform solution of the reaction products, resulting in soluble and insoluble fractions, which were separated and analyzed by FTIR spectroscopy. For example, the FTIR spectra of pristine PHBHHx and the two fractions of the S-05 sample are shown in Figure S6. The spectra indicate that the insoluble fraction, lacking the amide bond, comprised apolar hydroxyl-terminated chains, constituting the hydrophobic core of the nanoparticles. Conversely, the aminolyzed chains, containing the amide bond and the polar taurine salt derivative, exhibited solubility in the CHCl_3_/EtOH mixture. Their amphiphilic nature conferred surfactant activity, which was exploited to obtain PHBHHx nanoparticles. It is important to note that the separation of aminolyzed and nonaminolyzed fractions in this study was qualitative, without quantitative evaluation of fraction weights. The procedure aimed to verify the presence of the expected reaction products, as outlined in Figure 1. FTIR analysis confirmed the absence of aminolyzed species in the chloroform-soluble fraction; however, the presence of hydroxyl terminal moieties in the insoluble fraction cannot be definitively excluded.

### Nanoparticle Characterization

Unloaded and UA-loaded nanoparticles (N-X and N-X-UA) were prepared by using the emulsion drying technique. The aminolyzed samples dissolved in the reaction medium (EtOAc/EtOH) were directly added dropwise to water according to the procedure reported in the Experimental Section. UA loading was accomplished by dissolving the drug in a polymer EtOAc/EtOH solution. After removing organic solvents by heating the emulsion, DLS measurements were carried out in triplicate to evaluate the hydrodynamic diameter (*D*_H_) and zeta potential (ζ) of the formed nanoparticles. Both values were reported as mean values (*n* = 3) ± standard deviation in Figure 3A,B, respectively, as a function of the *M*_n_, which is indicative of hydrophobic domain dimensions and therefore could be considered an index of the surfactant activity. Additionally, SEM analysis has been conducted and reported in the Supporting Information (Figure S7).

**Figure 3:**
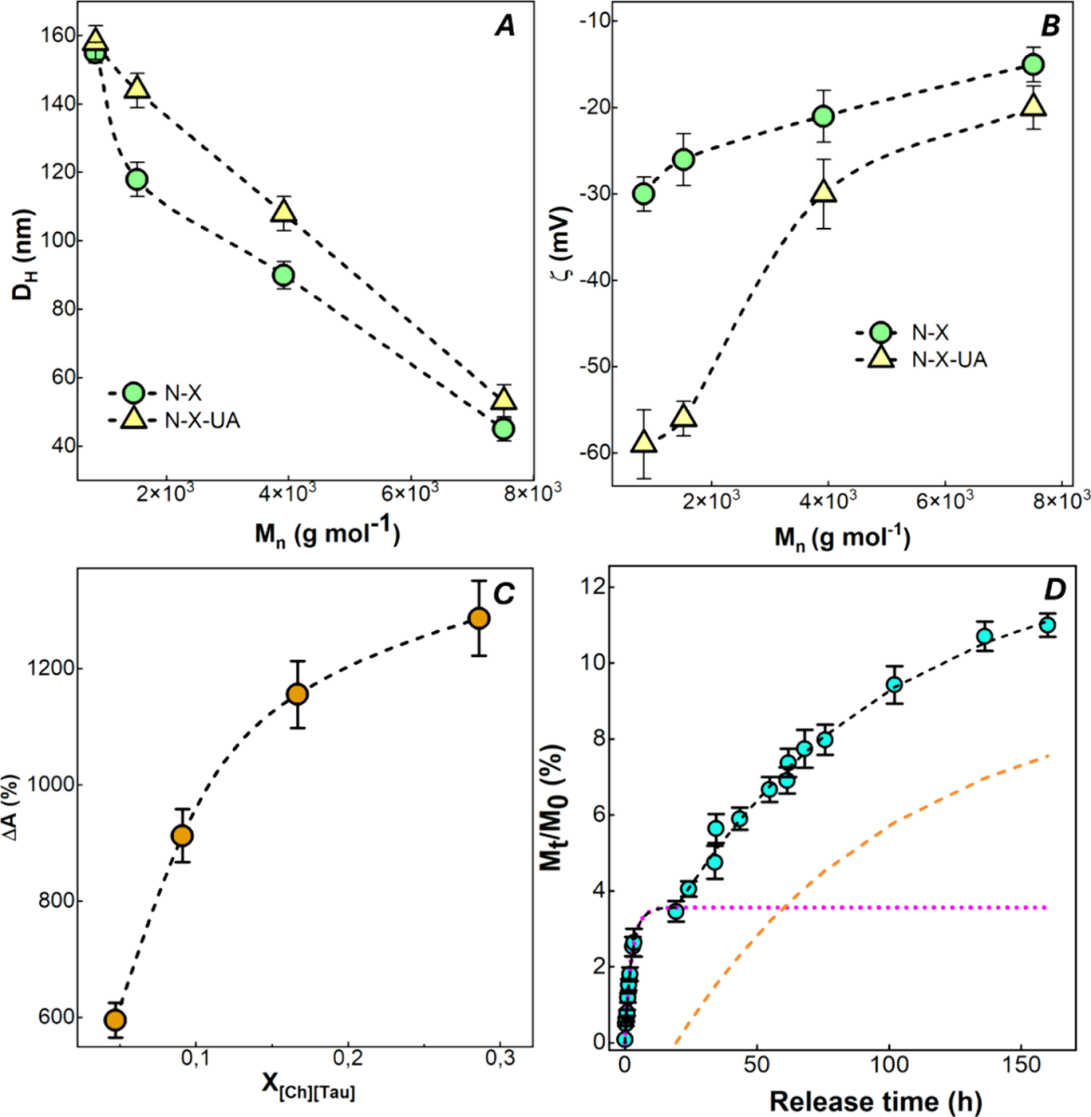
Nanoparticle characterization. (A) Hydrodynamic diameter *D*_H_ and (B) ζ potential of unloaded (N-X) and UA-loaded (N-X-UA) nanoparticles. (C) Apparent solubility Δ*A*% of UA-loaded nanoparticles (N-X-UA). (D) Cumulative drug release kinetics of N-17-UA nanoparticles in 0.9 wt %/v NaCl solution. The results are reported as mean values (*n* = 3) ± standard deviation. Fit curves of release data according to eq 7 (black line) and of the separate contributions of the first and second processes (dotted lines).

Figure 3A shows an inverse relationship between the nanoparticle size and molecular weight. The addition of usnic acid did not affect this correlation but induced a small increase in the hydrodynamic diameter (*D*_H_), with all other experimental parameters (stirring rate, reaction time, and initial polymer concentration) kept constant. This behavior is potentially counterintuitive. Indeed, more aminolyzed samples (corresponding to lower *M*_n_) are expected to form smaller nanoparticles due to a higher concentration of surfactant-active chains and improved hydrophilic/lipophilic balance. However, the increased hydrophilicity of the shorter chains could cause the nanoparticles to swell with water, thus increasing their volume.

The ζ measurements, presented in Figure 3 B, revealed negative values for all samples, consistent with the good colloidal stability of the nanoparticle dispersions. A slight, consistent decrease in the ζ absolute values was observed in unloaded samples as the *M*_n_ increased, likely attributable to the associated decrease in the SO_3_^–^ anion concentration. However, the incorporation of UA resulted in a marked ζ decrease, specifically for the N-17-AU and N-29-UA samples, characterized by the lowest *M*_n_. This observation suggests a migration of the polar terminal groups of the shorter chains from the nanoparticle core to the surface driven by unfavorable interactions with the hydrophobic UA molecules. This potential incorporation of the hydrophilic portion of the shorter chains within the nanoparticle structure may reduce the surfactant efficacy of the extensively aminolyzed polymer, thus contributing to the trend of increasing particle size observed in Figure 3A. PDI values for both N-X and N-X-UA are reported in Table 1. Noteworthily, according to *D*_H_ data reported in Figure 3A, drug-free nanoparticles are characterized by lower dimensions than those with drug. The presence of the drug favors the formation of larger nanostructures but with more uniform sizes.

**Table 1:** PDI Values for the N-X and N-X-UA Samples^a^

sample	PDI
N-29	0.424 ± 0.009
N-29-UA	0.159 ± 0.007
N-17	0.148 ± 0.009
N-17-UA	0.19 ± 0.008
N-09	0.24 ± 0.03
N-09-UA	0.211 ± 0.02
N-05	0.45 ± 0.01
N-05-UA	0.162 ± 0.008

a Data are reported as mean values (*n* = 3) ± standard deviation.

The determination of the apparent solubility (Δ*A*%) was used to evaluate drug encapsulation efficiency. Δ*A*% directly reflects the enhanced concentration of usnic acid achieved within the suspension relative to its maximum solubility in water. The results obtained for the various formulations, reported in Figure 3C, show that encapsulating the drugs into nanoparticles increased the UA apparent solubility from 596 to 1287%, which corresponds to an increase from 0.155 to 0.313 mg of loaded UA per mg of nanocarriers. Between the N-17-UA and N-29-UA samples, which show the highest UA content and ζ absolute value, N-17-UA was chosen for further biological activity experiments because of its lower nanoparticle dimension. The stability of the nanoparticle suspension was investigated by measuring its size over time. Specifically, the N-17 samples were suspended in water, PBS (0.1 M, pH 7.4), and physiological medium composed of a 0.2 mg mL^–1^ solution of albumin in PBS and analyzed by DLS over 1, 5, and 10 days. The results, expressed as the relative variation in hydrodynamic diameter with respect to the initial value (*D*_H_/*D*_H_^0^) and reported in Table S1 in the Supporting Information, indicate that the nanoparticles did not undergo aggregation up to the longest point analyzed time.

### Usnic Acid Release

The release in 0.9 wt %/v NaCl solution of UA loaded in N-17-UA samples, containing 0.283 mg of loaded UA per mg of nanoparticles, was performed by using the dialysis bag method. The cumulative UA release, expressed as *M*(*t*)/*M*_0_ (%), is reported as a function of the release time in Figure 3D. Figure 3D shows that approximately 12% of the drug was released within 160 h, consistent with the low UA aqueous solubility and its affinity for the hydrophobic nanoparticle core. The release kinetics exhibited an initial rapid release (burst effect) within approximately the first 5 h followed by a slower, sustained release up to the maximum experimental time. To investigate the overall mechanism, the drug release kinetics was modeled as the sum of two independent processes. At early times, the data are well described by first-order kinetics, according to eq 5. This model accounts properly for the slowdown in release observed after approximately 5 h. The subsequent release is slower than the initial burst and can also be adequately described by first-order kinetics, but with a time delay τ expressed by eq 6. Therefore, the overall release kinetics was modeled using eq 7. The fitting parameters in Table 2 show that the second release process is significantly slower than the initial burst release, as anticipated.

**Table 2:** Best Fit Parameters of the Eq 7 Interpolating Data of *M*(*t*)/*M*_0_ vs Release Time

*p*_1_ (%)	*k*_1_ (min^–1^)	*p*_2_ (%)	*k*_2_ (min^–1^)	τ (min)
3.5 ± 0.2	0.38 ± 0.03	9.3 ± 0.6	0.012 ± 0.001	19 ± 2

However, this slower phase makes a substantial contribution, accounting for 72% (*p*_2_ = 9.3%) of the total sustained release observed over the extended experimental period (*p*_1_ + *p*_2_ = 12.9%). The significant lag time indicates that the two distinct release phases exhibit minimal overlap. This biphasic release profile could be potentially advantageous for therapeutic antimicrobial formulations.^50^ This hypothesis was tested by biological characterization of the pristine and UA-loaded nanoparticles.

Indeed, the data of the long-term release process can be conveniently fitted also using the Korsmeyer–Peppas empirical model (eq 4) plus an additional term that accounts for the burst effect. The fitting process resulted in an *n* value of 0.6, which is close to the theoretical value of 0.5 predicted for Fickian diffusion. However, the Korsmeyer–Peppas model was unable to provide an estimate of the limit value of the total drug released during the second release phase.

### Antimicrobial Activity of N-17 and N-17-UA Nanoparticles

Bacterial infections are promoted by the spreading of planktonic cells, which ultimately colonize surfaces. During surface colonization, adherent bacteria undergo a phenotypic shift to a sessile state and begin secreting extracellular polymeric substances (EPSs), resulting in the formation of a biofilm. This biofilm serves as a protective barrier, shielding the bacteria from antimicrobial agents and other external stimuli. As the biofilm matures, external mechanical and physicochemical factors can lead to surface erosion and the subsequent dispersion of planktonic cells.

To evaluate the antimicrobial efficacy of unloaded N-17 and N-17-UA nanoparticles, both the planktonic and biofilm states of the bacteria were considered. Antimicrobial activity was assessed under static conditions to target planktonic bacteria and under dynamic fluidic conditions to investigate potential antibiofilm effects. The results are presented in Figure 4.

**Figure 4:**
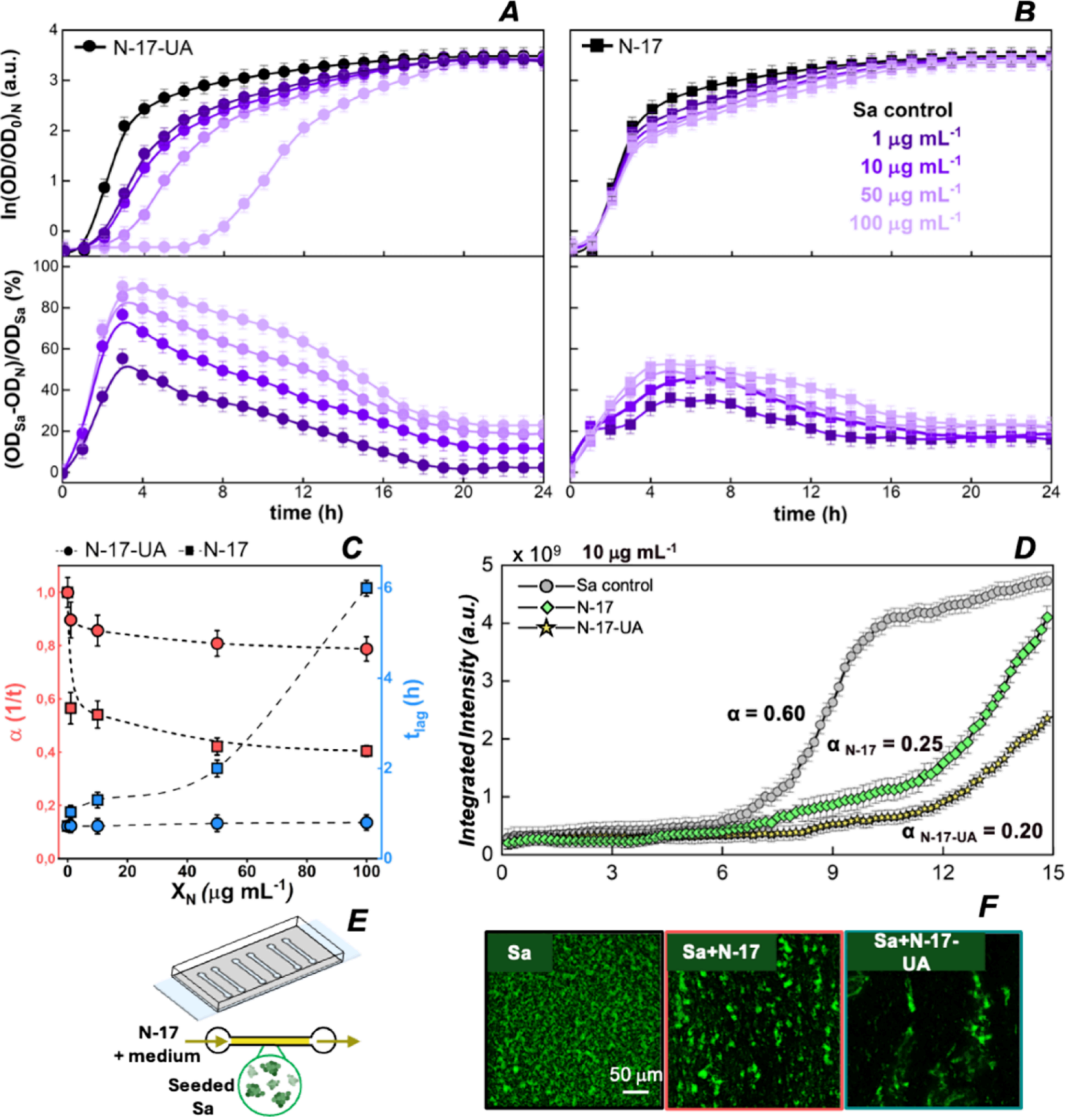
Antimicrobial activity of PHBHHx nanoparticles. Unloaded (N-17) and usnic acid loaded (N-17-UA) nanoparticles were tested against the Newman strain. (A) Top: growth curves of at varying concentrations of N-17-UA (1 μg mL^–1^ < *X*_N_ < 100 μg mL^–1^), reported as the natural logarithm of the normalized optical density measured at 600 nm (OD/OD_0_) every hour over 24 h. Bottom: percentage decrease in OD in the presence of N-17-UA nanoparticles, normalized to the OD of the control. (B) Top: growth curves of *S. aureus* obtained at different N-17 concentrations. Bottom: percentage decrease in OD in the presence of N-17 nanoparticles. Data are presented as mean ± SEM of three independent experiments. (C) growth rate (α) and lag time (*t*_lag_) as functions of nanoparticle concentration. (D) Integrated fluorescence intensity of GFP-tagged tracking biofilm formation in a microfluidic channel. Gray circles represent control biofilms, while green squares and yellow stars represent biofilms formed in the presence of a constant dose (10 μg mL^–1^) of N-17 or N-17-UA nanoparticles, respectively, flushed at a flow rate of 1.5 μL min^–1^ for 15 h. Each curve is annotated with its specific growth rate (α). Data are presented as mean ± SEM of three independent experiments. (E) Schematic representation of the microfluidic device used in the antibiofilm experiments. Bacteria were initially seeded at the bottom of the channel, incubated for 1 h, and subsequently flushed with either a control solution containing CB broth or suspensions of N-17 or N-17-UA nanoparticles in the CB medium. (F) Fluorescence optical microscopy images of biofilms formed within the microfluidic channel at 12 h. Images depict the control (left), biofilms in the presence of N-17 nanoparticles (center), and biofilms in the presence of N-17-UA nanoparticles (right).

The growth of a bacterial population in the planktonic state can be monitored by measuring the progressive turbidity of a bacterial suspension. Specifically, the optical density of the suspension at 600 nm (OD600) is measured. Under ideal conditions, bacterial cultures grow exponentially, and the number of cells (*N*) can be correlated to the logarithmic increase in the OD.

From a growth curve of ln­(OD) vs time (ln­(OD) calculated according to eq 8), the growth rate α can be determined from the slope of the curve. Additional insights, such as the lag time (*t*_lag_), can be obtained by identifying the final time point in the lower asymptote region, where no growth is detected before the exponential phase.

The growth curves shown in Figure 4B (top) illustrate the minimal impact on the growth rate and lag time of unloaded N-17 nanoparticles, with corresponding OD percentage values reported in Figure 4B (bottom) over 24 h, compared to a control (black squares).

In contrast, Figure 4A (top) shows that at concentrations as low as 1 μg mL^–1^, N-17-UA nanoparticles progressively reduced the growth rate of and extended the lag time, ultimately demonstrating antimicrobial activity by delaying bacterial replication. This indicates that the antimicrobial activity under planktonic conditions is predominantly mediated by the controlled release of usnic acid within the first 12 h, consistent with the release kinetics reported in Figure 3D. Once the bacterial cells entered the exponential growth phase, their population outnumbered the N-17-UA release rate, resulting in a converging stationary phase, albeit at slightly lower OD values compared to the control.

The growth rate (α) and lag time (*t*_lag_) as functions of nanoparticle concentration, derived from the growth curves in Figure 4A,B, are shown in Figure 4C. Remarkably, N-17-UA nanoparticles (red circles) at a concentration of 100 μg mL^–1^ reduced the growth rate of by over 50%, corresponding to an increase in duplication time from 36 to 80 min. Furthermore, the lag time (light-blue circles) scaled with N-17-UA concentration, reaching a maximum delay of 6 h at 100 μg mL^–1^. In contrast, unloaded N-17 nanoparticles had negligible effects on the growth rate (red squares) and lag time (light-blue circles). Interestingly, the antimicrobial efficacy of N-17-UA nanoparticles was primarily attributed to the controlled release of usnic acid, as free usnic acid dosed at equivalent encapsulation ratios (10:1 PHBHHx/UA) was less effective than N-17-UA nanoparticles (Figure S8). The results obtained from turbidimetry measurements were confirmed by performing a colony-forming unit assay at 24 h by incubating with the same concentrations of nanoparticles employed in the turbidimetry experiments and subsequent plating. The results are reported in Table S2. The antimicrobial activities of functionalized PHBHHx were further investigated by testing susceptibility to the ionic liquid [Ch]­[Tau]. [Ch]­[Tau] was dosed at the equivalent molar ratio of the polymer employed for the N-17 nanoparticle preparation. From the growth rates reported in Figure S9, it is possible to note a strong antimicrobial effect from 2 to 20 μg mL^–1^ with respect to the control, with almost no growth at 5 and 20 μg mL^–1^, contributing to the nanoparticles' antimicrobial activity.

Due to their dualistic nature, bacteria exhibit significant behavioral differences between their planktonic and sessile biofilm states. The controlled formation of bacterial biofilms can be studied using microfluidic systems, which replicate physiological growth conditions in confined environments while enabling multiplexing of various experimental conditions with minimal sample volumes. For this study, a microfluidic device with six rectangular channels (800 × 75 μm cross section) was fabricated by replica molding in polydimethylsiloxane (PDMS) from a silicon master. The PDMS channels were plasma-bonded to a glass slide and connected to a syringe pump. A suspension of cells was inoculated to fill the channels and incubated for 1 h to facilitate bacterial adhesion. The channels were then flushed with the CB medium (see the methods section) containing either N-17 or N-17-UA nanoparticles or the plain CB medium as a control. Biofilm formation was tracked by monitoring GFP fluorescence emitted by the cells using fluorescence optical microscopy. The results are shown in Figure 4D–F.

From the integrated fluorescence intensity (Figure 4D), the effects of N-17 and N-17-UA nanoparticles on biofilm formation can be compared. Under fluidic conditions, both nanoparticle formulations reduced the growth rate of biofilms, with N-17-UA nanoparticles demonstrating greater efficacy. However, in contrast to static conditions, unloaded N-17 nanoparticles also exhibited significant antibiofilm effects. This disparity is likely due to the amphiphilic nature of the PHBHHx nanoparticle matrix. Amphiphilic polymers may inhibit biofilm formation through various mechanisms, including altering the surface energy and charge to prevent initial bacterial attachment, disrupting the quorum sensing and microbial communication pathways essential for biofilm maturation, and penetrating and degrading the biofilm extracellular polymeric substance (EPS) matrix, thereby disrupting the biofilm integrity and disrupting bacterial membranes via the hydrophobic components of the polymer, leading to cell leakage and death.^51–53^

The observed antimicrobial activity may therefore result from a synergistic effect of the amphiphilic polymer carrier and the encapsulated agent. As shown in the fluorescence optical images (Figure 4F) and the videos reported in theSupporting Information, both N-17 and N-17-UA nanoparticles disrupted biofilm formation, demonstrating both biofilm inhibition and antimicrobial properties.

### In Vitro Cytotoxicity

To investigate the potential cytotoxic effects of nanoparticles, A549 epithelial cells were treated with incremental doses of nanoparticles (0–100 μg mL^–1^), and cytotoxicity and apoptosis were analyzed through a bioluminescent assay (ApoTox-Glo Triplex Assay Kit, Promega). As shown in Figure 5, A549 cells tolerated acute doses of both unloaded N-17 and usnic acid loaded N-17-UA nanoparticles up to 10 μg mL^–1^, displaying no sign of cytotoxicity at 24 h postadministration. When the concentration was increased to 50 μg mL^–1^, N-17 nanoparticles were well tolerated, with toxicity levels comparable to 10 μg mL^–1^, while N-17-UA nanoparticles had detrimental effects on cells. For N-17 nanoparticles, severe toxicity was observed only for the highest dose (100 μg mL^–1^).

**Figure 5:**
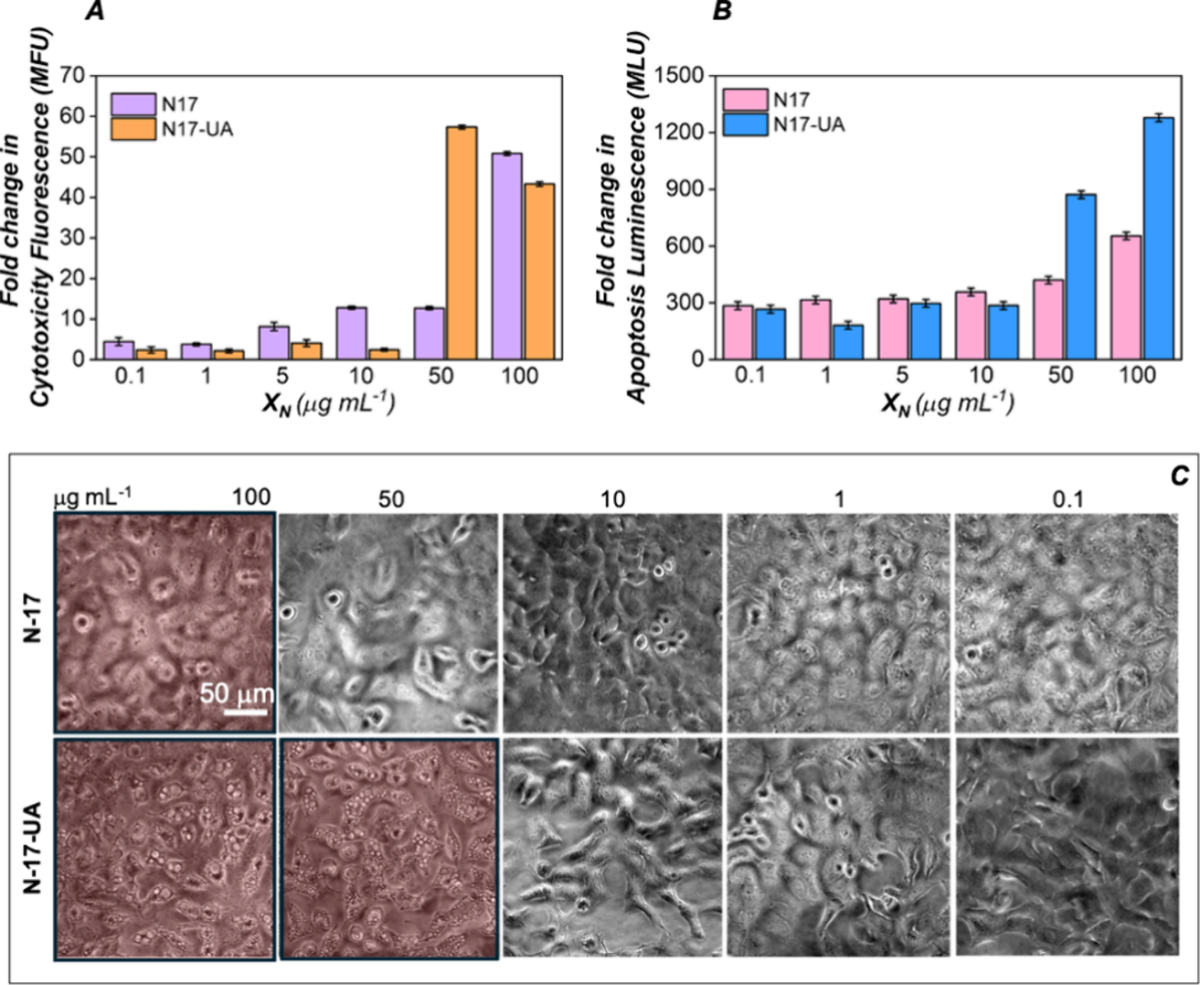
In vitro cytotoxicity and apoptosis. (A) Cytotoxicity and (B) apoptosis levels, expressed as mean fold change in fluorescence and luminance (MFU) relative to control, detected in A549 cells pretreated with increasing concentrations of N-17 and N-17-UA nanoparticles for 24 h. Data are presented as mean ± SEM. (C) Phase-contrast optical microscopy images of A549 cells seeded with different concentrations of N-17 and N-17-UA nanoparticles at 24 h.

Phase-contrast optical microscopy images presented in Figure 5C confirm the cytotoxic and apoptotic effects quantified with the bioluminescent assay ApoToxGlo.

N-17 nanoparticles caused an increase in cell cytotoxicity and apoptosis only for concentrations of 100 μg mL^–1^, when A549 cells started losing their plasma membrane integrity and leaked their cellular contents.

Instead, N-17-UA nanoparticles induce cell death at concentrations of 50 and 100 μg mL^–1^, with cells displaying several apoptotic bodies.

Notably, N-17-UA nanoparticles demonstrate potential as a promising noncytotoxic delivery agent at concentrations up to 10 μg mL^–1^ for systemic administration while still exhibiting significant antimicrobial activity. Higher doses could potentially be employed in antimicrobial formulations for external use or as coating agents for biomedical devices. To further assess the potential systemic application and biosafety of the N-17 and N-17-UA nanoparticles, a hemolysis study was performed (Experimental Section), and no significant impact on erythrocytes was found for the formulations employed in this study. The results are summarized in Table S3.

## Conclusions

The design of the proposed formulations was guided by consideration of key physicochemical properties of the selected chemicals, with a focus on sustainability, safety, ease, and efficacy of nanoparticle production. The mutual solubility of both [Ch]­[Tau] and PHBHHx in a specific organic solvent mixture allowed for the precise control of the aminolysis reaction, leading to the formation of tailored self-surfactant systems capable of stabilizing the resulting polymer suspension and enabling the production of nanoparticles with controlled sizes and drug loadings. The selection of safe reactants and solvents streamlined the experimental procedures, eliminating the necessity for complex and time-consuming purification processes that would otherwise be required to remove residual surfactant and potentially toxic solvents. The obtained nanoparticles loaded with UA showed a double-phase release composed of a rapid drug delivery and its slow elution for a longer time. The effectiveness of this behavior was assessed using biological assays, where the PHBHHx nanoparticles displayed antimicrobial efficacy on pathogenic and clinically relevant Newman, predicting an initial sufficiently high local drug concentration to kill planktonic bacteria. The subsequent sustained release was found to disrupt biofilm organization and inhibit subsequent regrowth by means of controlled physiological conditions achieved through microfluidics. Additionally, the unloaded nanoparticles were found to exert an intrinsic biofilm inhibitory activity due to the self-surfactant nature of the polymeric carrier possibly interfering with the charge distribution and quorum sensing of the biofilm components. The efficacy of usnic acid delivery was also confirmed by assessing the cytotoxicity on endothelial cells at different concentrations. Although for high dosages (>50 μg mL^–1^) cytotoxicity was induced, for dosages from 1 to 10 μg mL^–1^, relevant for systemic injections, the nanocarriers displayed no adverse effect on cells yet retained their antimicrobial activity. Overall, the purposed methodology allows for the sustainable preparation of antimicrobial amphiphilic polymers employable as encapsulants for a possible variety of lipophilic and bioactive molecules as potential alternatives to conventional antibiotic formulations.

## Experimental Section

### Materials

Poly­(3-hydroxybutyrate-*co*-3-hexanoate (PHBHHx, 11% mol 3-hydroxybutyrate 3HHx, Kaneka) was purified by precipitation, adding ethanol to the polymer ethyl acetate solution. Usnic acid (UA, Sigma-Aldrich), ethanol (EtOH, Carlo Erba), ethyl acetate (EtOAc, Sigma-Aldrich), taurine (Tau), and choline chloride (Ch-Cl) were used as received. The ionic liquid choline taurinate ([Ch]­[Tau]) was prepared according to the protocol previously reported.^35^ Briefly, an equimolar amount of KOH was added to a methanol solution of choline hydroxide. After the filtration of the formed KCl, a 20 mol % excess of a taurine solution in water was added. Then the solution was dried under a vacuum. The obtained [Ch]­[Tau] was dissolved in EtOH, and the taurine excess was filtered. Lastly, the alcoholic solution was dried under a vacuum, and [Ch]­[Tau] was obtained as a transparent viscous liquid.

### Aminolysis Reaction

PHBHHx is soluble in EtOAc at 65 °C and insoluble in EtOH. Vice versa, EtOH dissolves [Ch]­[Tau], which is insoluble in EtOAc. To conduct the aminolysis reaction in a homogeneous phase, preliminary experiments were carried out to find the right ratio between the two solvents to avoid polymer or taurine salt precipitation. An EtOAc/EtOH volume ratio of 2.5:1 was found to be the optimal mixture for the reaction.

After the complete polymer solubilization in EtOAc at 65 °C (2.5 wt/v %), a solution of [Ch]­[Tau] in EtOH was added dropwise under magnetic stirring. The reaction was conducted at 65 °C for 2 h under reflux. No increase in the reaction yield was observed for longer reaction times. The concentration of the ionic liquid in EtOH was adjusted to have molar fractions of aminolyzing agent (*X*_[Ch][Tau]_) of 0.286, 0.167, 0.091, and 0.07 (with respect to the mole of polymer repeating units) corresponding to 1 mol of [Ch]­[Tau] per 2.5, 5, 10, and 20 mol of PHBHHx repeating unit (RU). The stoichiometry and sample names of the aminolysis reaction products are reported in Table 2. At the end of the reaction, the solution was vacuum-dried, and the aminolysis product was analyzed. Moreover, fractionation of the reaction products was carried out by their solubilization in chloroform (0.5% wt/v) and precipitation by adding EtOH. After centrifugation, the solid and supernatant were vacuum-dried. The two fractions, soluble and nonsoluble in the CHCl_3_-EtOH mixture, were called S-X_s_ and S-X_ns_.

### Nanoparticle Preparation

Nanoparticles were obtained by an oil-in-water emulsion and subsequent solvent evaporation. Following aminolysis in EtOAc/EtOH, the reaction mixture was added dropwise to water under vigorous stirring (1500 rpm for 5 min). The resulting stable oil-in-water emulsion (o/w = 1:10 v/v) was maintained for 2 h at 65 °C under magnetic stirring to remove EtOAc.

To prepare drug-loaded nanoparticles, UA was dissolved in EtOAc (0.5% w/v) and added to the reaction medium at the end of aminolysis. In Table 3, the sample codes of the aminolyzed polymer, unloaded, and UA-loaded nanoparticles according to the *X*_[Ch][Tau]_ used in the reactions are reported_._

**Table 3:** Sample Codes of Aminolysis Products of Unloaded and UA-Loaded Nanoparticles according to the *X*_[Ch][Tau]_ Used in the Reactions

PHBHHx/[Ch][Tau] (mol:mol)	*X*_[Ch][Tau]_	aminolysis product	nanoparticles	UA loaded nanoparticles
1:0	0	S-0		
20:1	0.047	S-05	N-05	N-05-UA
10:1	0.091	S-09	N-09	N-09-UA
5:1	0.167	S-17	N-17	N-17-UA
2.5:1	0.286	S-29	N-29	N-29-UA

### Characterization

#### Nuclear Magnetic Resonance (NMR) Spectroscopy

^1^H and decoupled ^13^C NMR spectra were collected on a Bruker Avance NEO 400 Nanobay. All spectra of low- and high-molecular-weight (MW) compounds were acquired using 32 and 1024 scans with a delay of 2 or 6.5 s, respectively. Each NMR tube was prepared by dissolving [Ch]­[Tau] in D_2_O and PHBHHx and its modifications in CDCl_3_ at a concentration of 10 mg mL^–1^_._

#### Fourier Transform Infrared Spectroscopy

The products of the PHBHHx aminolysis reaction were analyzed by FTIR spectroscopy using a Thermo Nicolet 6700 instrument (Thermo Scientific, Waltham, MA, USA). The spectra of aminolysis reaction products, cast from chloroform solution on SeZn disk, were acquired by coadding 200 scans in the 4000–650 cm^–1^ range at a resolution of 4 cm^–1^. The extent of functionalization has been determined by calculating the amide index, which is the ratio between the intensity of the amide II peak located at 1579 cm^–1^ and that of the band at 1453 cm^–1^, (δ_as_ CH_3_), not involved in the reaction and not affected by sample crystallinity.^42^

#### Gel Permeation Chromatography (GPC)

The S-X samples were dissolved in chloroform at a concentration of 0.6% w/v, filtered (Whatman 0.2 μm PTFE syringe filters), and analyzed by a gel permeation chromatography (GPC) equipped with a pump (Jasco PU-4180), a guard column, and two linear columns in series (TSKgel G6000-HHR TSKgel GMHHR-H) maintained at 40 °C through a column oven (Jasco CO-4060) and a refractive index detector (Jasco RI-4030). Chloroform was used as an eluent at a flow rate of 1 mL/min. The detector was set at 35 °C. Polystyrene standards (1.3 × 10^3^–3.05 × 10^6^ g mol^–1^) were used to calibrate the system. Deconvolution of overlapped peaks was carried out by using the Gaussian curve (eq 1):

$$I_{i}(t)=I_{i}^{0}e^{-\frac{(t-t_{i}^{\max}{)}^{2}}{{\mathrm{FWHH}}_{i}^{2}}}$$ 1

where *t* is the elution time and the parameters related to the sum of two curves (*I*_tot_ = *I*_1_ + *I*_2_), that is, peak intensity *I*_*i*_^0^, elution time at the maximum *t*_*i*_^max^, and full width at half-maximum fwhh, were determined through a best-fit program. The integrated area of the Gaussian function (*A*_G1_) at the highest molecular weight with respect to the overall chromatogram (*A*_tot_) was calculated as eq 2:

$$G1\%=\frac{A_{G1}}{A_{\mathrm{tot}}}\times 100$$ 2

#### UV–Vis Spectroscopy

UV–vis spectrophotometry was used to evaluate the amount of UA encapsulated within nanoparticles, measuring the absorption at 286 nm. UV spectra were acquired by a diode-array spectrophotometer (Hewlett-Packard 8452A) in the range of 190–820 nm at a resolution of 2 nm.

Drug encapsulation efficiency was estimated through the apparent solubility (Δ*A*%) determination according to the following formula (eq 3):

$$\Delta A\%=\frac{\Delta A}{A_{0}}\times 100=\frac{\left(A-A_{0}\right)}{A_{0}}\times 100$$ 3

where *A* is the absorbance of UA-loaded nanoparticle suspension and *A*_0_ is the absorbance values of usnic acid in a solution obtained in the same condition used for nanoparticle preparation but without the polymer. Then Δ*A*% represents the increase of the concentration of the drug due to its encapsulation in the nanoparticle suspension with respect to the concentration of the drug possibly solubilized in water during nanoparticle formation. To remove nanostructure scattering, we subtracted a spectrum of the suspension of unloaded nanoparticles from that of loaded nanoparticles. From the Δ*A*% values, the concentration of usnic acid within the nanoparticles, expressed as milligrams of loaded UA per milligram of nanocarriers, was evaluated.

#### Usnic Acid Release

The dynamic dialysis method has been used to evaluate usnic acid cumulative release.^43,44^ A 5 mL portion of the UA-loaded nanoparticle suspension (0.1 mg mL^–1^) was put into a dialysis bag (cutoff 13,000) and immersed in 90 mL of a physiological solution (NaCl 0.9%) in sink conditions. Aliquots of 2 mL were sampled at periodic intervals and analyzed with UV–vis spectroscopy. The same aliquot of fresh NaCl 0.9% was replaced to maintain a constant volume in the system.

The cumulative release fraction was expressed as *M*(*t*)/*M*_0_, where *M*_0_ is the amount of drug in the nanosystems at the beginning and *M*(*t*) is the drug released at time *t*. Each release experiment was carried out by sampling two microparticle aliquots from each of the two independent preparations. The cumulative release results are reported as the mean value ± maximum deviation. Korsmeyer–Peppas was applied to model the release mechanism. The Korsemeyer–Peppas equation, applicable for the first 60% of the release of the drug,^45^ is expressed as eq 4:

$$\frac{M\left(t\right)}{M_{0}}=Kt^{n}$$ 4

where *M*(*t*)/*M*_0_ represents the fractional released drug, *t* is the time, *K* is the release constant related to structural features of the nanocarriers, and *n* is the transport exponent (adimensional), linked to the drug release mechanism (Fickian diffusion or non-Fickian diffusion).

Alternatively, the overall drug release kinetics was modeled as the sum of two independent processes. At early times, the data were described by first-order kinetics according to the following eq 5:

$$\frac{M\left(t\right)}{M_{0}}=1-\exp\left(-k_{1}t\right)$$ 5

where *k*_1_ is the first-order rate constant, related to drug diffusion through the nanoparticles and the drug dissolution rate. The subsequent release was described by a modified first-order kinetics following eq 6:

$$\frac{M\left(t\right)}{M_{0}}=1-\exp\left[-k_{2}\left(t-\tau \right)\right]$$ 6

where *k*_2_ is the rate constant and τ is a time delay of the second release process. Therefore, the overall release kinetics was modeled using the following equation (eq 7):

$$\frac{M\left(t\right)}{M_{0}}=p_{1}\left[1-\exp\left(-k_{1}t\right)\right]+p_{2}\left\{1-\exp\left[-k_{2}\left(t-\tau \right)\right]\right\}$$ 7

where *p*_1_ and *p*_2_ represent the fractions of drug released in the first and second processes, respectively, with *p*_2_ = 0 when *t* < τ.

#### Scanning Electron Microscopy (SEM)

The formation of nanoparticles was further confirmed by scanning electron microscopy using a field emission scanning electron microscope (AURIGA, Zeiss, Jena, Germany). Nanoparticle aqueous suspensions were cast on a silica plate and dried. Prior to the measurements, the samples were sputtered with gold.

#### Antimicrobial Activity

The antimicrobial activity of unloaded N-17 and usnic acid loaded N-17-UA nanoparticles was assessed by evaluating the effect of nanoparticle concentration on the growth rate of the fluorescent GFP-tagged (Sa) Newman strain under static conditions. Bacterial suspensions were prepared by inoculating cultured colonies grown on agar plates into 3 mL of Columbia broth (CB, BD DIFCO, 30 g L^–1^) and incubating at 37 °C for 4 h with shaking at 250 rpm. Following incubation, the bacterial suspension was diluted to a final concentration of 10^–3^ cells mL^–1^. Subsequently, 300 μL of the bacterial suspensions was transferred into a 48-well plate and incubated with varying concentrations of N-17 and N-17-UA nanoparticles, ranging from 1 to 100 μg mL^–1^. To mimic the encapsulated amount of UA in the nanoparticle formulations (0.5% w/v), free UA was also tested at equivalent concentrations. Bacterial growth over 24 h was monitored by measuring the optical density at 600 nm (OD600) hourly using a plate reader (Biotek Synergy H4 Hybrid). The variation of ln­(OD) measured over hours was used to estimate the growth rate of the bacterial population according to the following equation (eq 8):

ln(ODOD0)≈α(t−t0) 8

where OD_0_ is the optical density of the suspension at time *t* = 0 and α is the growth rate. A colony-forming unit assay was performed by incubating with N-17 and N-17-UA nanoparticles at a concentration on 1, 10, 50, and 100 μg mL^–1^ and free UA at a concentration of 1:10 to the loaded and unloaded nanoparticles. Incubation was performed for 24 h, and serial dilutions were performed up to 10-7. Fifty microliters of the bacterial suspension was plated on LB-agar plates and incubated overnight. Colony counting was performed at a dilution of 10-7 under all conditions.

The influence of N-17 and N-17-UA nanoparticles on the biofilm formation was further investigated. Bacterial suspensions (10 cells mL^–1^) were inoculated into a series of rectangular microfluidic channels (800 × 75 μm cross section) and incubated for 1 h. Following incubation, N-17 and N-17-UA nanoparticle suspensions in a CB medium along with a CB medium control were injected at a flow rate of 1.5 mL min^–1^ for 18 h. GFP fluorescence signals from cells were live-imaged using fluorescence optical microscopy every 15 min.

#### In Vitro Cytotoxicity Cell Culture

A549 cells were cultured in Dulbecco’s modified Eagle’s medium (DMEM) (Sigma-Aldrich, St. Louis, MO, USA) with 10% fetal bovine serum (FBS) (Sigma-Aldrich, St. Louis, MO, USA), 1% penicillin–streptomycin (Pen–Strep 10,000 U/mL, Lonza, Basel, CH), 1% ultraglutamine (200 mM, Lonza, Basel, CH), and 1% sodium pyruvate (100 mM Lonza, Basel, CH). Cultures were maintained at 37 °C in a humidified environment with 5% carbon dioxide.

#### Cell Viability, Cytotoxicity, and Caspase 3/7 Activity Assay

To test the biocompatibility of nanoparticles, A549 cells (5 × 105 cells mL^–1^ in 100 μL) were seeded in black 96-well plates (ViewPlate-96 Black, PerkinElmer, Waltham, Massachusetts, USA) and treated with N-17 and N-17-UA ranging from 1 to 100 μg mL^–1^.

Viability, cytotoxicity, and caspase-3/7 activities were measured 24 h after nanoparticle treatment using the ApoTox-Glo Triplex Assay kit (Promega, Madison, WI, USA) according to the manufacturer’s instructions. Cell viability and cytotoxicity, determined by live/dead-cell protease activity, were assessed by measuring fluorescence for viability at 400 and 505 nm and for cytotoxicity at 485 and 520 nm. Caspase-3/7 activity was analyzed by measuring luminescence with a microplate reader (Synergy H4 Hybrid Multi-Mode Microplate Reader, Biotek Synergy H4 Hybrid).

#### Erythrocyte Hemolysis

Blood samples were collected from healthy donors within the IRCCS Humanitas Research Hospital. The erythrocytes were immediately separated by centrifugation at 2000*g* for 5 min and washed three times with 4 vol of a normal saline solution. Erythrocytes collected from 1 mL of blood were resuspended in 10 mL of normal saline. Immediately thereafter, 2.5 mL of 2% (w/v) dispersions of the nanoparticles and mixtures thereof in saline were incubated with 0.1 mL of the erythrocyte suspension. Incubations were carried out at 37 °C with gentle tumbling of the test tubes. After 1 h of incubation, the samples were centrifuged for 5 min at 2000*g*. The absorbance of the supernatant was measured at 415 nm to determine the number of cells undergoing hemolysis. Hemolysis induced with double-distilled water was taken as a positive control. The hemolysis ratio was calculated as follows (eq 9):

hemolysisrate=(Dt−Dnc)/(Dpc−Dnc) 9

where Dt, Dnc, and Dpc represent the absorbances measured at 415 nm for the sample, negative control in PBS, and positive control in distilled water, respectively.

## Supplementary Material









## Notes

Number-average molecular weight (*M*_n_) and amide index of amidated PHBHHx as a function of reaction time (Figure S1); ^1^H NMR spectrum of [Ch] [Tau] (Figure S2); (A) ^1^H NMR and (B) ^13^C NMR spectrum of poly­(hydroxy butyrate)-*co*-(hydroxy hexanoate) (S-0) (Figure S3); ^1^H NMR comparison between S-0 and S-29 (Figure S4); ^13^C NMR comparison between S-0 and S-29 (Figure S5); FTIR spectroscopic characterization of fractionated reaction products (Figure S6); SEM image of N-17 (Figure S7); stability data of N-17 particles expressed as the relative variation in hydrodynamic diameter with respect to the initial value (*D*_H_/*D*_H_^0^) (Table S1); colony-forming units per mL (CFU/mL) of in the presence of N-17, N-17-UA nanoparticles, and free UA (Table S2); N17 and N17-UA nanoparticle hemolysis ratio (Table S3); growth curves of in the presence of free usnic acid (Figure S8); growth curves of in the presence of free [Ch]­[Tau] (Figure S9); and usnic acid cytotoxicity in A459 cells pretreated with increasing concentrations of UA mimicking the amount encapsulated in PHBHHx carriers (0.5% w/v) for 24 h (Figure S10) (PDF)
Biofilm formation in fluidic conditions for control of in the presence of N17 and N17-UA nanoparticles (Video S1) (ZIP)
Biofilm formation in fluidic conditions for control of in the presence of N17 and N17-UA nanoparticles (Video S2) (ZIP)
Biofilm formation in fluidic conditions for control of in the presence of N17 and N17-UA nanoparticles (Video S3) (ZIP)

## Notes

Gianluca Forcina: CNRS, CEA/LETI Minatec, Laboratoire des Technologies de la Microélectronique (LTM), Université Grenoble Alpes, 38000 Grenoble, France
