# Supplementary material for: Self-Surfactant Poly-3hydroxybutyrate-co-3hydroxyhexanoate (PHBHHx) for the Preparation of Usnic Acid Loaded Antimicrobial Nanoparticles Using Nontoxic Chemicals
Source: ACS Appl Bio Mater. 2025 Jun 19;8(7):6109–20. doi: 10.1021/acsabm.5c00676 (PMC12284855; doi:10.1021/acsabm.5c00676)
Supplement: Supplementary file 1 [file mt5c00676_si_001.pdf]

Supporting Information

Self-surfactant poly-3hydroxybutyrate-co-3hydroxyhexanoate (PHBHHx) for the preparation of usnic acid-loaded antimicrobial nanoparticles using non-toxic chemicals

Sara Alfano [a], Lorenzo Ceparano [a], Benedetta Brugnoli [a], Gianluca Forcina [a], Luca Pellegrino [b*], Francesca Cecilia Lauta [b], Roberto Rusconi [b,c], Iolanda Francolini [a], Antonella Piozzi [a], Andrea Martinelli [a]*

[a] Sapienza University of Rome, Department of Chemistry, P.le A. Moro 5, 00185 Rome, Italy

[b] Department of Biomedical Sciences, Humanitas University, Via Rita Levi Montalcini 4, 20072 Pieve Emanuele, Italy

[c] IRCCS Humanitas Research Hospital, Via Manzoni 56, 20089 Rozzano, Italy

CORRESPONDING AUTHOR(S): Sara alfano (sara.alfano@uniroma1.it); Luca Pellegrino (luca.pellegrino@humanitasresearch.it)

SUPPORTING INFORMATION

Table of contents:

Figure S1. Number average molecular weight (M_n) and amide index of amidated PHBHHx as a function of reaction time.

Figure S2 ^{1}H-NMR spectrum of [Ch] [Tau]

Figure S3. A) ^{1}H-NMR and B) ^{13}C-NMR spectrum of poly (hydroxy butyrate)-co-(hydroxy hexanoate) (S-0)

Figure S4. ^{1}H-NMR comparison between S-0 and S-29.

Figure S5. 13C-NMR comparison between S-0 and S-29.

Figure S6. FTIR spectroscopic characterization of fractionated reaction products

Figure S7 SEM image of N-17

Table S1. Stability data of N-17 particles expressed as the relative variation in hydrodynamic diameter with respect to the initial value (D_H/D_H^0)

Table S2. Colony forming units per mL (CFU/mL) of S. aureus in presence of N-17, N-17-UA nanoparticles and free UA.

Table S3. N17 and N17-UA nanoparticles hemolysis ratio**.**

Figure S8. Growth curves of *S. aureus* in presence of free usnic acid

Figure S9. Growth curves of *S. aureus* in presence of free [Ch][Tau]

Figure S10. Usnic acid cytotoxicity in A459 cells pretreated with increasing concentration of UA mimicking the amount encapsulated in PHBHHx carriers (0.5% w/v) for 24 h

Video S1-S2-S3 Biofilm formation in fluidic conditions for control of *S. aureus* in presence of N17 and N17-UA nanoparticles

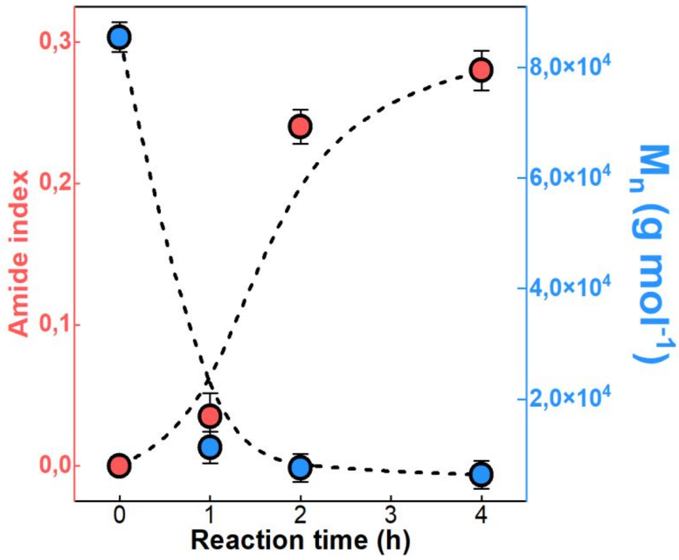

Figure S1 – Characterisation of functionalized PHBHHx at different reaction times Amide index and Mn values of functionalized PHBHHx obtained at different reaction times.

In Figure S2, ^{1}H-NMR spectrum of [Ch] [Tau] is shown. As previously reported by Deng et al. [10.1515/gps-2017-0009], the peak at 4.11- 4.03 ppm is related to the methylene groups next to the -OH, whereas the triplet at 3.56-3.49 ppm can be attributed to the methylene groups next to quaternary ammonium group, both related to the Ch motif. Instead, the peak at 3.21 ppm regards the methyl groups linked to the nitrogen, while the singlet at 3.08 ppm is attributed to the methylene groups of the Tau motif.

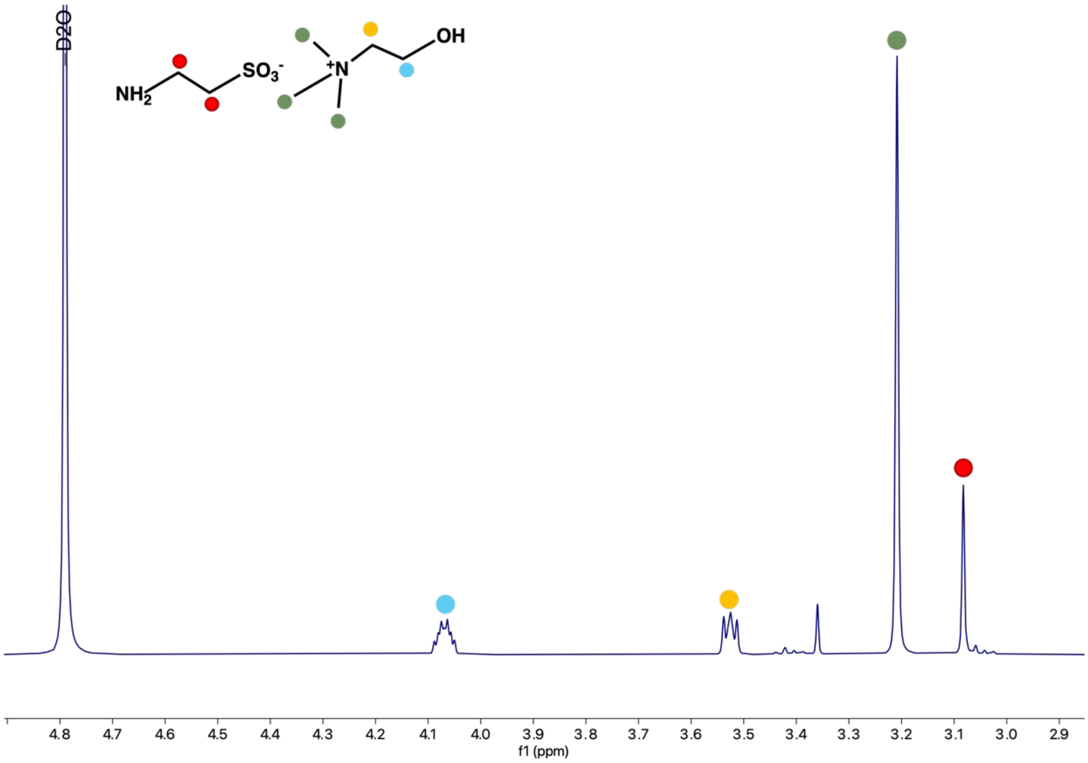

Figure S2. ^{1}H-NMR spectrum of [Ch] [Tau]

^{1}H NMR (400 MHz, D_2O) δ 4.11 – 4.03 ppm (m, 2H, CH_2OH), 3.56 – 3.49 ppm (m, 2H, $CH_2N^+(CH_3)_3$), 3.21 ppm (s, 9H, CH_3), 3.08 ppm (s, 4H, $CH_2CH_2SO_3^-$) (MeOH traces at ca. 3.34 ppm).

To identify the presence of amide groups in both the pure (S-0) and aminolyzed (S-29) samples , ^{1}H and ^{13}C spectra were recorded.

In Figure S2, the ^{1}H-NMR spectrum of PHBH (S-0) is reported. The triplet at 0.91 ppm is related to the methyl group of the side-chain of the hexanoate block (H), whereas the methyl group of the hydroxy butyrate block is downshifted (1.26 ppm). The multiplet at 1.56 ppm can be attributed to the methylene groups of the H block. Instead, the methylene peaks of the polymer main chain are at 2.89-2.21 ppm while the protons of the methine groups of both are downshifted at 5.24 ppm.

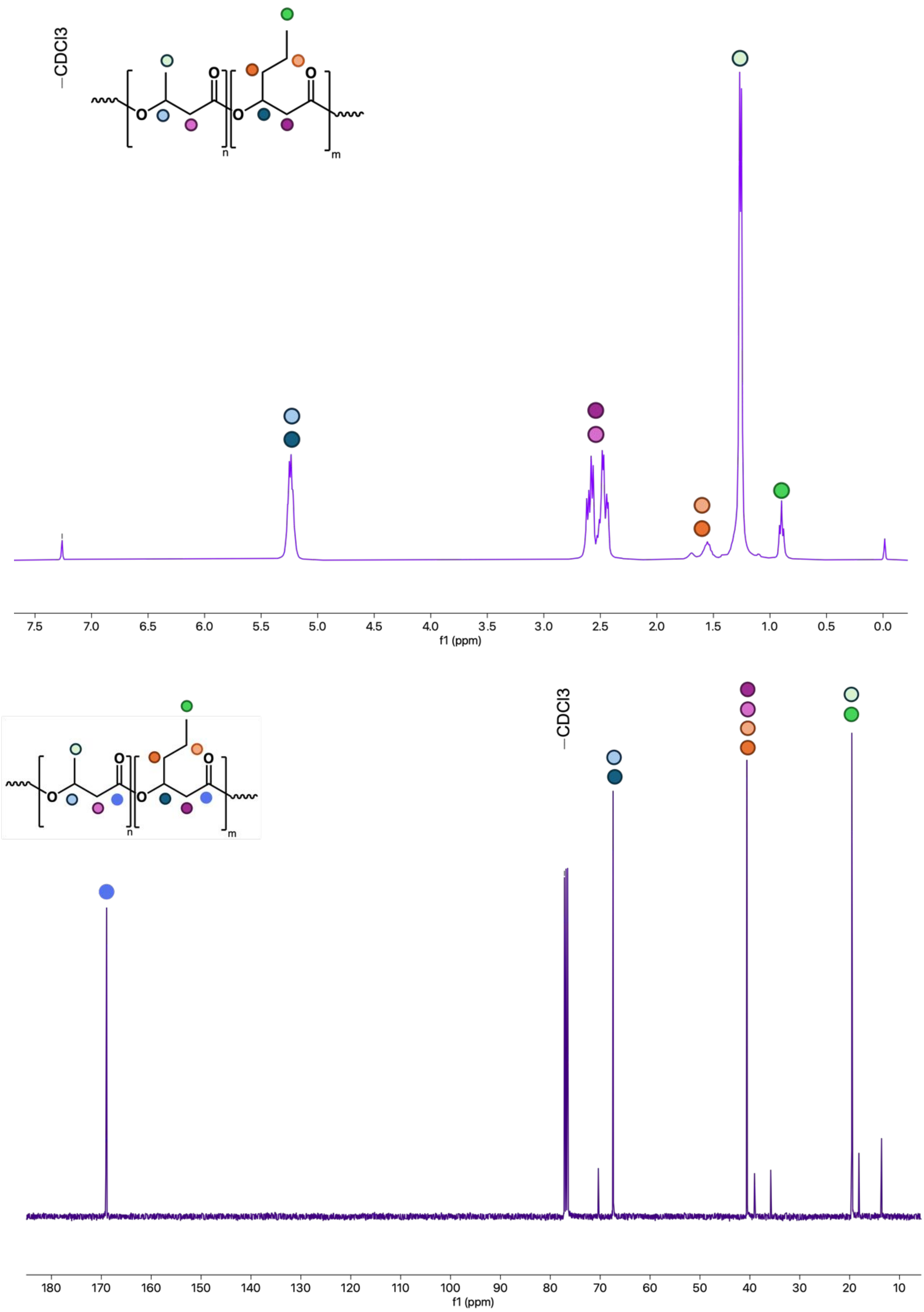

Figure S3. A) ^{1}H-NMR and B) ^{13}C-NMR spectrum of poly (hydroxy butyrate)-co-(hydroxy hexanoate) (S-0)

^{1}H NMR (400 MHz, $CDCl_3$) δ 5.24 ppm (h, 2H, CH(B) and CH(H)), 2.89 – 2.21 ppm (m, 4H, CH_2(B) and CH_2(H)), 1.56 ppm (h, 1H, CH_2(H)), 1.26 ppm (d, 6H, CH_3(B)), 0.90 ppm (t, 3H, CH_3(H)); ^{13}C NMR (101 MHz, $CDCl_3$) δ 169.58 ppm (C=O(B) and C=O(H)), 68.80-67.29 ppm (CH(B) and CH(H)), 42.52-41.23 ppm (CH_2(B) and CH_2(H)), 22.10- 18.30 ppm (CH_3(B) and CH_3(H)).

Differences can be observed among the proton spectra of S-0 and S-29 (Figure S4), since a singlet at 3.4-3.3 ppm may be related to the methylene group next to the sulphate moiety of Tau.

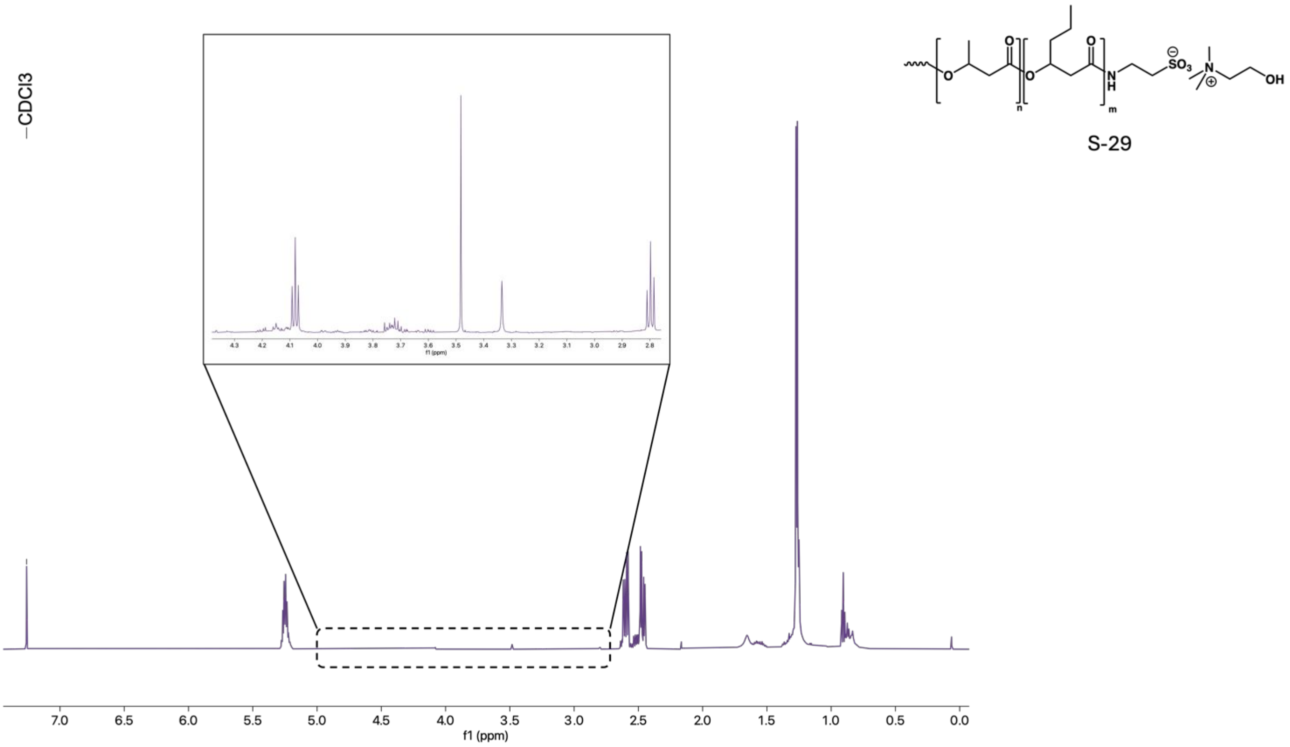

Figure S4. ^{1}H-NMR comparison between S-0 and S-29.

Additionally, differences could be observed in the carbon spectra (Figure S5). The aminolysis occurrence was further confirmed by the presence of new peak around 32 ppm that could be assigned to the carbons next to the amide groups, not present in the pure polymer, [10.1515/gps-2017-0009].

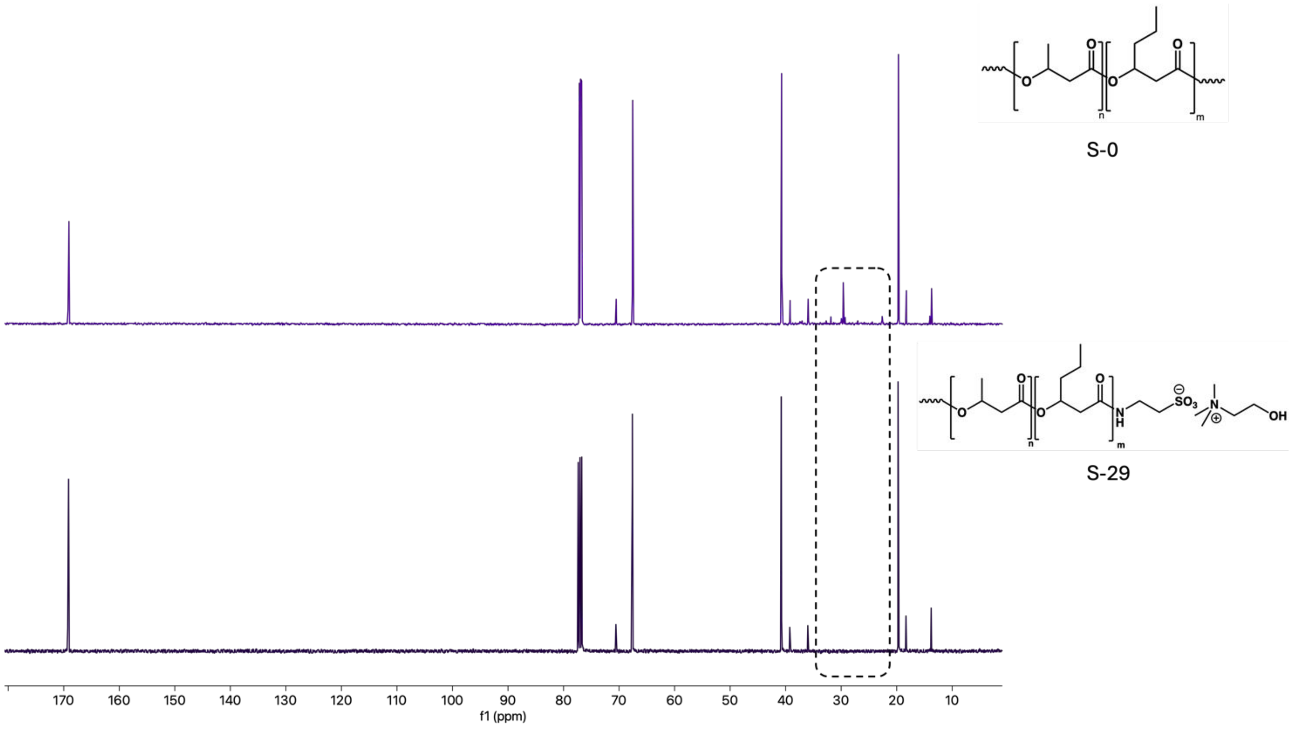

Figure S5. ^{13}C-NMR comparison between S-0 and S-29.

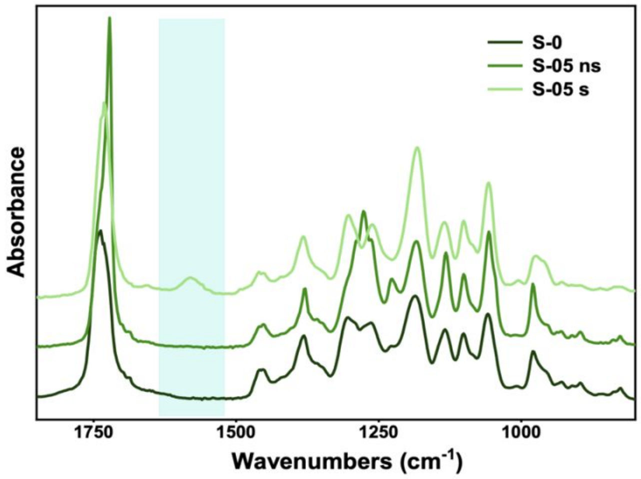

Figure S6 – Spectroscopic characterization of fractionated reaction products FTIR spectra of pristine PHBHHx and the soluble (S-05s) and non-soluble (S-05ns) fractions of the S-05 sample. The soluble fraction comprises highly functionalized polymer chains (corresponding to product 1 reported in Figure 1), while the non-soluble fraction does not show typical amide peaks (corresponding to product 2 reported in Figure 1).

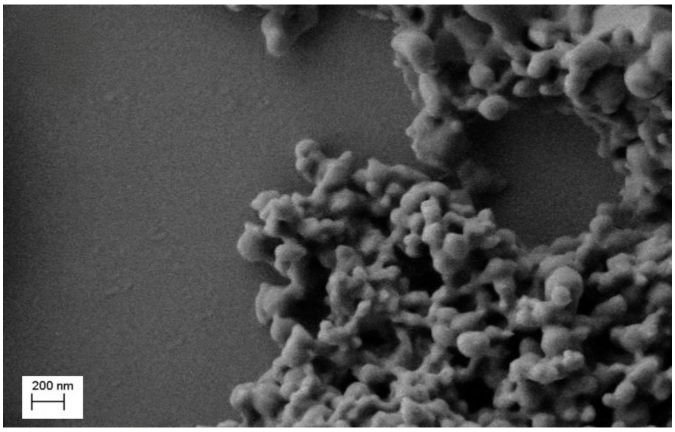

Figure S7. SEM image of N-17

Table S 1. Stability data of N-17 particles expressed as the relative variation in hydrodynamic diameter with respect to the initial value (D_H/D_H^0)

Time (d)	Water D_H/D_H^0	PBS	Albumin
1	1.262 ± 0.0269	1,013 ± 0.0269	1,059 ±0.0276
5	0.999 ± 0.0236	0,964 ±0.0262	1,051 ±0.0268
10	1.036 ± 0.0240	0,998 ±0.0267	1,052 ±0.0268

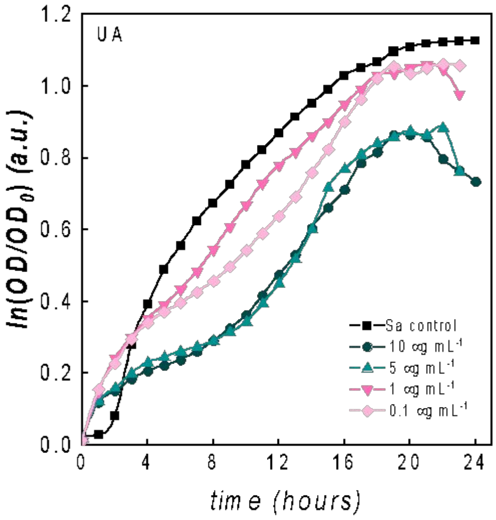

Figure S8 Growth curves of S aureus in presence of free usnic acid. UA was dosed mimicking the amount encapsulated in PHBHHx carriers (0.5% w/v) for 24 h.

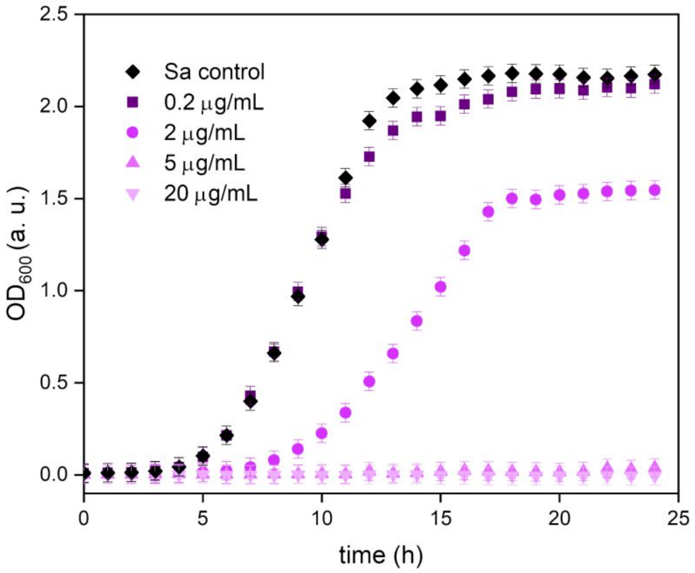

Figure S9 Growth curves of S aureus in presence of free [Ch][Tau]. The ionic liquid was dosed according to its molar ratio with respect to the PHBHHx, i.e. 1:5 [Ch][Tau]:PHBHHx. Data are expressed as mean ± SEM over three different experiments.

Table S2. Colony forming units per mL (CFU/mL) of S. aureus in presence of N-17, N-17-UA nanoparticles and free UA. Experiments were run in triplicate with colony counting performed at 24 h. Statistical analysis was performed via one-way ANOVA with a $p<0.001$. Sa control CFU/mL = 3.7×10^{10}

UA (µg mL^{-1})	0.1	1	5	10
CFU/mL	2.38×10^{10}	1.9×10^{10}	6×10^{10}	3×10^{10}
N-17 (µg mL^{-1})	1	10	50	100
CFU/mL	2.08×10^{10}	1.8×10^{10}	2×10^{10}	2×10^{10}
N-17-UA (µg mL^{-1})	1	10	50	100
CFU/mL	2.14×10^{10}	1.6×10^{10}	1×10^{10}	9×10^{10}

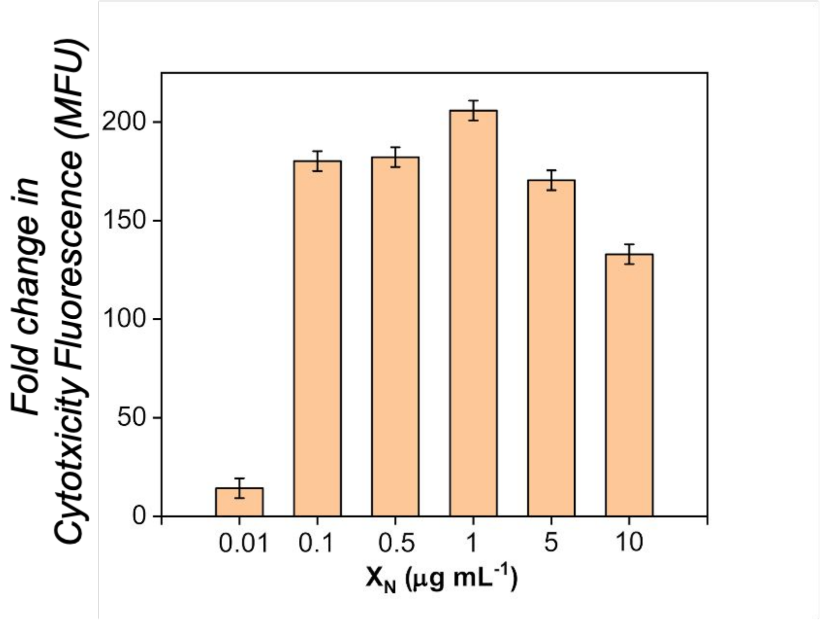

Figure S10 Usnic acid cytotoxicity**.** Cytotoxicity levels, expressed as fold change in mean fluorescence intensity relative to control (MFU), detected in A549 cells pretreated with increasing concentration of UA mimicking the amount encapsulated in PHBHHx carriers (0.5% w/v) for 24 h.

Table S3 N17 and N17-UA nanoparticles hemolysis ratio**.** The erythrocytes hemolysis ratio was calculated as (Dt - Dnc) / (Dpc - Dnc) where Dt, Dnc and Dpc represent the absorbances measured at 415 nm for the sample, negative control in PBS and positive control in distilled water respectively.

Hemolysis Degree Dt

			**N-17 (µg/mL)**		
**D_{nc} (PBS)**	**1**	**10**	**50**	**100**	**D_{pc} (H20)**
0.0007 ±0.0002	0.0003 ± 0.0005	0.0033 ± 0.0003	0.0066 ± 0.0021	0.0093 ± 0.0027	1 ± 0.0064
			**N-17-UA (µg/mL)**		
	**1**	**10**	**50**	**100**	
	0.0022 ± 0.0017	0.0041 ± 0.0015	0.0052 ± 0.0093	0.0065 ± 0.0093	
